# Diagnoses, infections and injuries in Northern Syrian children during the civil war: A cross-sectional study

**DOI:** 10.1371/journal.pone.0182770

**Published:** 2017-09-08

**Authors:** Gerlant van Berlaer, Abdallah Mohamed Elsafti, Mohammad Al Safadi, Saad Souhil Saeed, Ronald Buyl, Michel Debacker, Atef Redwan, Ives Hubloue

**Affiliations:** 1 Department of Emergency Medicine, Universitair Ziekenhuis Brussel, Brussels, Belgium; 2 Research Group on Emergency and Disaster Medicine, Vrije Universiteit Brussel, Brussels, Belgium; 3 Department of Emergency Medicine, Hamad Medical Corporation, Doha, Qatar; 4 Qatar Red Crescent, Medical Office for Turkey and Syria, Turkey Mission, Doha, Qatar; 5 Department of Paediatric Cardiology, Hamad General Hospital, Doha, Qatar; 6 Department of Public Health, Biostatistics and Medical Informatics Research Group, Vrije Universiteit Brussel, Brussels, Belgium; 7 Department of Critical Care, Faculty of Medicine, Zagazig University, Zagazig, Egypt; Johns Hopkins Bloomberg School of Public Health, UNITED STATES

## Abstract

**Background:**

The civil war in Syria including the deliberate targeting of healthcare services resulted in a complex humanitarian emergency, seriously affecting children's health. The objectives of this study are to document diagnoses and disease categories in Northern Syrian children after four years of conflict, and to document infectious diseases and injuries in this vulnerable population.

**Methods:**

In a prospective cross-sectional observational sample study conducted in May 2015, healthcare workers registered demographics, comorbidities, and diagnoses (categorised according to the International Classification of Diseases version 10) in children visited at home and in internally displaced persons camps in four Syrian governorates.

**Results:**

Of 1080 filled-out records, 1002 were included. Children originated from Aleppo (41%), Idleb (36%), Hamah (15%) and Lattakia (8%). Median age was 6 years (0–15; IQR 3–11), 61% were boys, 40% were younger than 5 years old. Children suffered from respiratory (29%), neurological (19%), digestive (17%), eye (5%) and skin (5%) diseases. Clinical malnutrition was seen in 4%, accidental injury in 3%, intentional injury in 1%, and mental disorders in 2%. Overall, 64% had features of infectious diseases (OR 0.635; CI 0.605–0.665). Most common comorbidities were chronic respiratory diseases (14, malnutrition (5%), acute flaccid paralysis (5%), and epilepsy (4%). Logistic regression analysis indicated that the risk for children to have communicable diseases was higher in Aleppo than in Idleb (OR 1.7; CI 1.2–2.3), Hamah (OR 4.9; CI 3.3–7.5), or Lattakia (OR 5.5; CI 3.3–9.3). Children in Aleppo and Lattakia were more at risk to be injured than in Idleb (OR 5.6; CI 2.1–14.3), or in Hamah (OR 5.9; CI 1.4–25.6), but more often from intentional violence in Lattakia. Mental problems were more prominent in Hamah.

**Conclusions:**

Four years far in the conflict, 64% of the studied children in four Northern Syrian governorates suffer from infections, mostly from respiratory, neurological and digestive origin, while 4% was injured or victim of intentional aggression. Substandard living conditions and the lack of paediatric healthcare put Syrian children at risk for serious infections, epidemics and morbidity, and ask for urgent international humanitarian relief efforts.

## Introduction

### Background/Rationale

According to the WHO the Syrian civil war ignited in 2011 led to the worst humanitarian disaster yet in the 21st century, with over 250000 verifiable violent deaths, 300000 injured, and more than 100000 missing, although the real toll is estimated much higher [1–3]. Many Syrians were relocated several times: seven million to internally displaced persons (IDP) camps, another five million to refugee camps (RCs) in neighbouring countries, and more than a million undertook dangerous journeys towards Europe [4–5].

Among these war victims are many children with over 17000 documented deaths, mostly due to bombing, shrapnel and gunshot wounds [1,6,7]. Children account for 18% of all injured Syrians, and those who survived may be left with physical and mental disabilities [6,8]. Very young children are forcibly being recruited by terrorist groups to serve as fighters or human shields [3]. Half of all displaced people are minors, more than 300000 Syrian children have been born as refugees in the meantime, and over 15000 unaccompanied minors have crossed the Syrian borders [3]. Children residing in IDP camps or RCs are at increased risk for illness, malnutrition, abuse, trafficking and exploitation because of compromised access to protective shelter, safe water, nutritious food, and paediatric healthcare [4,9].

Prior to the war, diseases and causes of mortality and morbidity in Syrian children up to 15 years old underwent a slow transition from acute communicable and mainly water-borne diseases, towards more chronical and typical Western non-communicable diseases, like hypertension, diabetes, overweight and asthma [10–12]. Adverse outcomes–expressed as disability adjusted life years–mostly resulted from infectious, maternal, perinatal and nutritional disorders, with diarrhoeal diseases, prematurity, low birth weight, and lower respiratory infections as leading causes. These were followed by non-communicable diseases, mainly due to congenital anomalies, anaemia and nutritional deficiencies (primarily iodine, proteins and iron), unintentional injuries, cardiovascular, endocrine and neuropsychiatric conditions, and chronic respiratory conditions like asthma [11–14]. After five years of armed conflict, destruction of healthcare facilities, and the exodus of two-thirds of all healthcare workers, life expectancy in Syria dropped by 12 years [2,10,15]. Violence- and war-related injuries became the first cause of mortality and morbidity [13]. Eight million Syrian children are in urgent need of humanitarian and medical assistance today, some of whom were originally Iraqi refugees that had fled their country during the 2003 invasion, now forcibly displaced again [3,8,16]. Immunisation rates–a prominent paediatric health care denominator–dropped to less than half [17,18]. This triad of violence, imploded healthcare, and reduction in immunisation coverage provides suitable conditions for re-emergence and outbreaks of infectious diseases that were formerly under control. Hepatitis, measles, leishmaniasis, multi-drug-resistant tuberculosis, typhoid and even polio–which had not been seen in the Middle East for 20 years–are now spreading across the land and into neighbouring and other countries [2]. It is expected that following violence and resettlement to new environments, one in four victimized children will suffer from physical, and two-thirds from mental health problems severe enough to affect their life [19–21].

Global health literature covering the Syrian situation is scarce, and did not yet deliver sufficient epidemiologic data to provide a comprehensive and complete overview [1,2,4,6,8,11,17,22–26]. Some studies discussed the health problems of Syrian refugees in neighbouring countries, or asylum seekers in European countries, but they vary widely in methodology and points of interest, and most are expert opinions or undocumented statements [7,16,19,27–33]. Some grey reports discuss the burden of mortality and morbidity, but also lack to document specific diagnoses. Under-researched areas are infectious disease control, surveillance of displaced populations, non-communicable disease, and specifically the health of Syrian children [34,35].

A prerequisite to adopting any evidence-based approach in humanitarian assistance is to assemble solid evidence from results of empirical studies [36]. Collecting reliable data will always be difficult in crisis situations, as healthcare providers prioritise treating patients over documenting and control groups are usually not available [2,30,35].

### Objectives

The objectives of this study are to document diagnoses, disease categories, and comorbidities in Syrian children after four years of conflict, and to document infectious diseases and injuries in a vulnerable paediatric population.

## Materials and methods

### Study design

A prospectively designed cross sectional observational sample analysis was performed on medical records, obtained and collected by Qatar Red Crescent (QRC) on May 21st and 22nd 2015, four years after the start of the civil war. The study protocol was approved by the Ethical Committee of the Universitair Ziekenhuis Brussel, Belgium. (O.G. 016): approval number B.U.N. 143201524794. A data sharing and research collaboration agreement was signed between QRC and the researchers. Qatar Red Crescent obtained permits to perform medical work in Syria in 2011, and approval to collect medical data as necessary for scientific studies, conducted in compliance with the relevant international regulations and laws.

### Setting

QRC is active in all seven Northern Syrian governorates with 173 healthcare professionals, largely experienced in independent monitoring of polio immunisation campaigns and sampled cluster household surveys. Since 2011 QRC runs several humanitarian relief projects in accordance with the United Nations High Commissioner for Refugees (UNHCR) supporting displaced people with basic needs like drinking water, sanitation and healthcare, as well inside as around the Syrian borders. This study was conducted in the context of the ninth polio immunisation campaign by QRC. Before the start of the study, a data sheet (available in English and Arabic) was designed focusing on demographic and health data of children. A group of 30 data collectors was selected by QRC/WHO experts and received a training course provided by the research group of this study, explaining the concept, methodology, process, and forms of the study, as well as how to debrief victims and how to deal with difficult humanitarian issues. The data collectors then visited households at home and in IDP camps in the Northern Syrian governorates of Aleppo, Idleb, Hamah and Lattakia. The data sheets were pooled and sent to the QRC office in Gaziantep, Turkey.

### Participants

For each immunisation campaign, QRC visits households, temporary shelters, and small local health centres within geographically predefined clusters (governorate districts) used in former campaigns in the four Northern governorates. In order to meet the predefined target total number of 1080 children, within each visited district a simple random sample of Syrian children was selected in the context of the ninth QRC polio immunisation campaign. Every child younger than 15 years old was eligible for inclusion. If the child was too young to answer some of the questions, these were answered by the guardian of the child. In accordance with the Ethical Committee of the Universitair Ziekenhuis Brussel, Belgium, oral informed consent from the child and the guardian was obtained and documented before the interview and medical exam. There has been no other filtering or restriction in selection of samples for this study.

### Variables

By means of a prospectively designed medical data sheet (see S1 File), the data collectors registered the child’s age, gender, habitat details regarding displacement, water and sanitation, access to health care, and the child’s medical record: clinical nutrition state, vaccination state, up to four medical acute diagnoses, comorbidities and malnutrition- or infection-related diseases. Diagnoses were based on anamnesis and physical examination as the QRC team had little access to laboratory or imaging diagnostic capability. One primary and all secondary diagnoses were recorded according to a list of 41 possible diagnoses in a template used in previous humanitarian operations by a number of large governmental and non-governmental organisations, and which was adapted from case descriptions in the WHO "Communicable disease control in emergencies" field manual and the Sphere Project Handbook [30,37–39]. Diagnoses were categorised beforehand according to the International Classification of Diseases (ICD-10) [40]. All patients with clinical signs of local or generalised infection were classified in a subgroup of "infectious diseases", as diagnosed clinically (see Table 1, case descriptions marked with *).

**Table 1:** Diagnostic categories, primary diagnoses and case descriptions.

CATEGORY	n	%	PRIMARY DIAGNOSIS	n	%	CASE DESCRIPTIONS
NO DIAGNOSIS	11	1.1	no medical diagnosis	11	1.1	social problem, attention seeker
RESPIRATORY	291	29.0	upper respiratory tract infection	139	13.9	ear, nose, throat, sinus, larynx infections, flu (upper ARI)*
			lower respiratory tract infection	90	9.0	dyspnoea, and raised respiratory rate, signs of lower ARI*
			asthma exacerbation	62	6.2	wheezing and/or respiratory oppression
EYE & ADNEXA	48	4.8	eye disorder	48	4.8	eye infection* and irritation
DIGESTIVE	167	16.7	watery diarrhea/abdominal	77	7.7	loose stools, vomiting, abdominal pain, intestinal parasitosis*
			bloody diarrhoea	20	2.0	loose stools with visible blood (suspicion of dysentery)*
			malnutrition	42	4.2	clinical, weight/height >70% or MUAC <110/160 (child/adult)
			cholera	3	0.3	severe dehydrating diarrhoea/confirmed case non-endemic area*
			jaundice	25	2.5	acute onset of icterus (skin, conjunctivae, urine)
NEUROLOGICAL	192	19.2	suspected meningitis	73	7.3	fever and clinical signs of meningeal irritation*
			acute flaccid paralysis	57	5.7	child ≥1 limb(s) (incl. Guillain Barré) or any age polio suspicion*
			CVA, headache, convulsions	62	6.2	headache, convulsions, stroke, coma
GENITOURINARY	39	3.9	sexual transmittable disease	0	0.0	suspected STD, vaginal infections with fluor, genital infection*
			urinary tract infection	38	3.8	dys-, alg-, pollakisuria, with(out) fever, flank pain, or + dipstick*
			gynaecological disorder	1	0.1	irregular menses, breast problems, vaginal bleeding, abortion
PERIPARTUM	8	0.8	neonatal illness	6	0.6	newborns with problems
			neonatal tetanus	2	0.2	neonate not sucking/crying normally, rigidity, convulsions*
			healthy new-born baby	0	0.0	healthy baby <3 weeks old
SKIN	47	4.7	skin infection	47	4.7	redness, pain, abcedation with signs of local infection*
OTHER	102	10.2	surgical cases other than trauma	4	0.4	hernias, swollen testicles, cysts, haemorrhoids,…
			fever of unknown origin	11	1.1	>37,5°C axillary or > 38,0°C rectal, without specific diagnosis*
			malaria	1	0.1	confirmed or suspected malaria, simple or serious*
			measles	2	0.2	fever and clinical suspicion of measles (vaccinated or not)*
			clinical anaemia	48	4.8	history, pallor, weakness
			diabetes	8	0.8	diabetes as main problem, or crisis/ketoacidosis
			other	13	1.3	diagnoses not classified elsewhere
			intoxication	0	0.0	suspected or confirmed substance abusus
			neoplasm	15	1.5	suspected or confirmed oncological disease
MENTAL	17	1.7	mental disorder	17	1.7	PTSD, insomnia, stress, suspicious aspecific complaints
VIOLENCE	7	0.7	trauma from aggression	3	0.3	trauma due to intentional individual injury including rape
			CBRN	4	0.4	injury from chemical/biological/radiological/nuclear attacks
INJURY	30	3.0	accidental trauma	26	2.6	accidental trauma from incident, accident (non-violent trauma)
			acute wounds	4	0.4	non-intentional acute skin wounds, burns
MUSCULOSKELETAL	5	0.5	musculoskeletal disorder	5	0.5	non-traumatic pain (muscles, back, pelvic belt, joints), rheuma
CIRCULATORY	18	1.8	hypertension/cardiac disorder	18	1.8	symptomatic hypertension, palpitations, angina pectoris
FATALITY	2	0.2	resuscitation	0	0.0	any condition requiring resuscitation of vital functions
			death	2	0.2	deceased patients
FOLLOW-UP CASES	18	1.8	follow-up wound dressings	2	0.2	follow-up of wound dressings and injections / vaccinations
			follow-up fractures & casts	14	1.4	follow-up old fractures & casts cases
			follow-up other	2	0.2	follow-up of chronic illness, other cases
**TOTAL**	**1002**	**100**	**Total**	**1002**	**100**	** **
*subanalysis*			infectious cases	636	63.6	all cases with features of infection (marked with *)
*subanalysis*			trauma cases	37	3.7	all cases with trauma diagnoses

ARI: acute respiratory infection, MUAC: middle upper arm circumference, CVA: cerebrovascular accident, incl: including, STD: sexually transmittable disease, PTSD: post-traumatic stress disorder, CBRN: chemical-burns-radiological-nuclear incidents, +: positive

All data were anonymised according to the Helsinki Declaration [41]. The collected records were scanned and analysed by qualified auditors to remove incomplete forms. The data were compiled into an interface especially designed for this study by the QRC data entry team, which included a data entry manager, an auditor, and analysts. Finally, the dataset was sent to the researchers and once more revised.

### Statistical methods

Children with missing or unreadable data were excluded. Descriptive statistics for discrete outcome variables were presented as frequencies and proportions (n; %), and for quantitative variables (age, number of children) as median, range, and interquartile range (IQR). The analyses were broken down for age (<5 and ≥5years old) and region (4 governorates).

Pearson's chi-square test was performed to identify factors associated with the health problems by using 'infectious disease' and 'injury' as outcome variables, and by using origin (governorate), age category (<5 and ≥5years old), and gender as associated factors. Crude Odds Ratios were adjusted using multiple logistic regression analysis. Overall goodness of fit was evaluated using a likelihood ratio-test and the Hosmer–Lemeshow test. Model quality was evaluated using the Nagelkerke’s R^2^. Analyses were carried out by using SPSS v23.0 (IBM Corp, Armonk, NY). All tests were performed using an α-level of 0.05.

## Results

### Descriptive data

A total of 1002 children were included in the study. For some of the descriptive data the number of participants differs from the total included population, depending on availability of information or on relevance: for vaccination state only infants over the age of the first vaccination, and for access to education only school-aged children (≥6 years old) were considered.

The included children were recruited in Aleppo (n = 413; 41%), Idleb (n = 360; 36%), Hamah (n = 147; 15%) and Lattakia (n = 82; 8%). More than half were male (n = 612; 61%), females accounted for 39% (n = 390). Median age was six years old (range 0–15; IQR 3–11). Children younger than five accounted for 40% (n = 398), children older than five for 59% (n = 596). The exact age was missing for seven children. Almost 20% (n = 196) of all children were displaced to IDP camps at the time of the study. This was more often the case in Lattakia (n = 38/82; 46%) and Hamah (n = 49/147; 33%), than in Aleppo (n = 61/413; 15%) and Idleb (n = 48/360; 14%). Additionally, 52 (6%) were displaced to constructions or homes, other than their own house. Of 13% (n = 132) one or both parents were deceased or missing. More than half of all school-aged children (n = 451/883;51%) had no access to education. Children lacked access to safe drinking water sources in 8% (n = 80), to appropriate sanitation facilities in 14% (n = 135), to regular nutrition in 15% (n = 147), and to paediatric healthcare providers in 63% (n = 632). Immunisation state was inadequate in 30% (n = 298/985), with missing immunisations for measles (n = 126;12%), hepatitis B (n = 99;10%), tetanus/pertussis/diphtheria (n = 96;10%), polio (n = 78;8%), and/or tuberculosis (n = 77;8%).

### Outcome data

**Fig 1:**
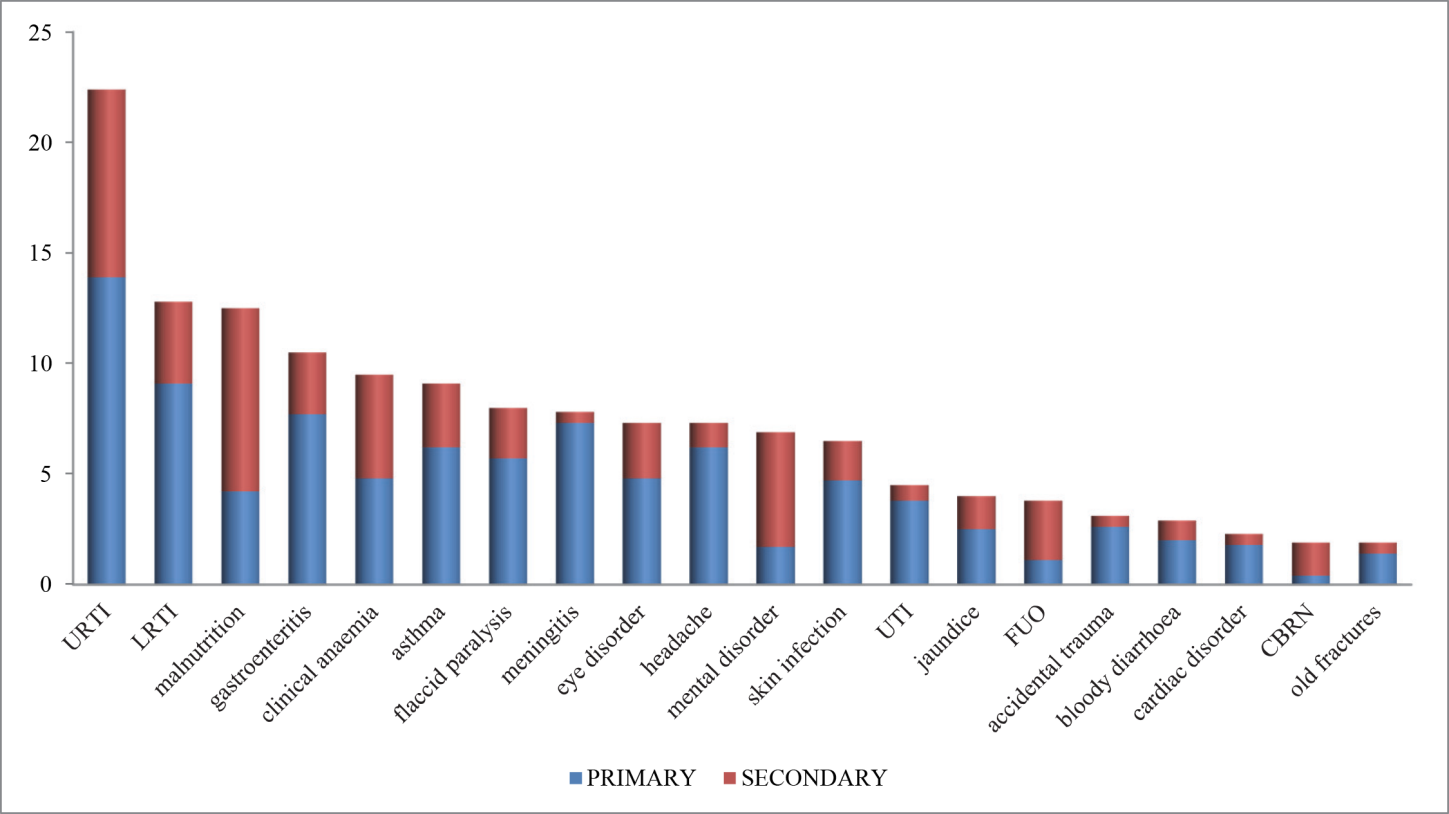
Most common (primary and secondary) diagnoses observed in Syrian children (%). URTI: upper respiratory tract infection; LRTI: lower respiratory tract infection; UTI: urinary tract infection; FUO: fever of unknown origin; CBRN: lesions from chemical, biological, radiological and nuclear attacks.

As represented in Fig 1 and Table 1, the most common single primary diagnoses consisted of upper (14%) and lower (9%) respiratory tract infections, watery diarrhea (8%), suspected meningitis (7%), acute asthma (6%), convulsions (6%), and acute flaccid paralysis (6%). Thirty-seven children (4%) were injured, of whom seven (1%) had been victim of intentional violence: four had lesions of chemical attacks (0.4%). Two children died during the time of the study.

Regarding secondary supplementary diagnoses at the time of the study, another 122 children suffered from upper respiratory infections (9%), diseases related to malnutrition (8%), clinical anaemia (5%), lower respiratory infections (4%), watery (3%) or bloody diarrhea (3%), eye infections (3%), and acute asthma crises (3%). In addition to the 37 children with injury as primary diagnosis, 27 (3%) children had injuries as secondary diagnosis, including lesions originating from chemical attacks (n = 15 or 2%). An additional 52 (5%) had mental health disorders as secondary diagnosis.

**Fig 2:**
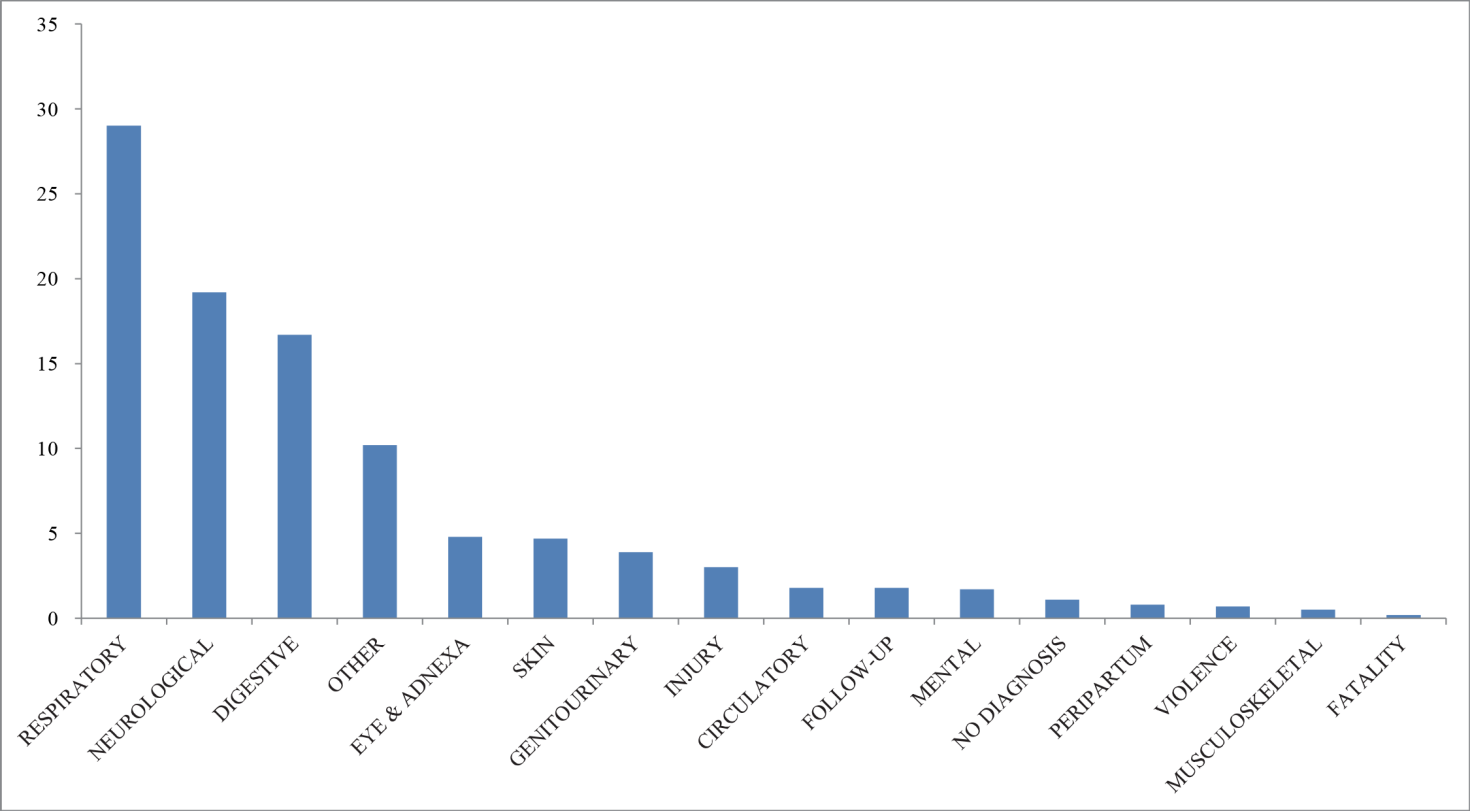
Distribution of ICD diagnosis categories in the total population (%).

An overview of encountered primary diagnostic ICD-10 categories is given in Fig 2. The most frequent were respiratory (29%), far ahead of neurological (19%) and digestive (17%) diagnoses. More than 4% of children had been victim of violence or injury, and 2% of children suffered from mental problems.

Table 2 and S1 Fig illustrate regional differences between governorates. In Aleppo, respiratory diseases (34%), eye problems (6%), and injury (7%) were more common than in other governorates. In Aleppo and Idleb, children suffered proportionally more from digestive diseases (respectively 21% and 19%) than in Hamah or Lattakia (7% and 5%). Physical aggression towards children was reported more often in Lattakia (5%). Neurological disorders were more predominant in Hamah and Lattakia (28% and 29%), and mental disorders were more prominent in Hamah (4%) than in the other regions.

**Table 2:** Relative proportions of primary diagnosis categories broken down per governorate (n,%).

	Aleppo	Idleb	Hamah	Lattakia	Total
No diagnosis	2 (0.5)	4 (1.1)	5 (3.4)	0 (0)	11 (1.1)
Respiratory	141 (34.1)	101 (27.9)	29 (19.7)	20 (24.4)	291 (29.0)
Eye & adnexa	26 (6.3)	16 (4.5)	4 (2.7)	2 (2.4)	48 (4.8)
Digestive	85 (20.6)	68 (18.9)	10 (6.8)	4 (4.9)	167 (16.7)
Neurological	45 (10.9)	82 (22.8)	41 (27.9)	24 (29.3)	192 (19.2)
Genitourinary	21 (5.1)	13 (3.6)	3 (2.0)	2 (2.4)	39 (3.9)
Peripartum	2 (0.5)	2 (0.6)	0 (0)	4 (4.9)	8 (0.8)
Skin	23 (5.6)	16 (4.5)	7 (4.8)	1 (1.2)	47 (4.7)
Other	24 (5.8)	41 (11.4)	26 (17.7)	11 (13.4)	102 (10.2)
Mental	8 (1.9)	3 (0.8)	6 (4.1)	0 (0)	17 (1.7)
Violence	2 (0.5)	1 (0.3)	0 (0)	4 (4.9)	7 (0.7)
Injury	25 (6.1)	2 (0.6)	1 (0.7)	2 (2.4)	30 (3.0)
Musculoskeletal	2 (0.5)	2 (0.6)	1 (0.7)	0 (0)	5 (0.5)
Circulatory	2 (0.5)	5 (1.4)	7 (4.8)	4 (4.9)	18 (1.8)
Fatality	0 (0)	1 (0.3)	0 (0)	1 (1.2)	2 (0.2)
Follow-up	5 (1.2)	3 (0.8)	7 (4.8)	3 (3.7)	18 (1.8)
**TOTAL**	**413 (100)**	**360 (100)**	**147 (100)**	**82 (100)**	**1002 (100)**
*Infectious diseases*	314 (76.0)	235 (65.3)	57 (38.8)	30 (36.6)	636 (63.6)
*Trauma (injury + violence)*	27 (6.5)	3 (0.8)	1 (0.7)	6 (7.3)	37 (3.7)

Clinical features of infection were found in 636 of all 1002 children (64%), with an even higher proportion of 70% (n = 280/398) in children younger than five. Tables 3 and 4 indicate that infections were more likely to be present in Aleppo and Idleb, than in Hamah and Lattakia (p<0.0001).

**Table 3:** Identifying factors associated with infectious diseases and injury by Pearson's Chi-square (n,%).

	Associated Factors					Significant	p-value
**Infectious**	Governorate	Aleppo	Idleb	Hamah	Lattakia	+	<0.0001
		314 (76.0%)	235 (65.3%)	57 (38.8%)	30 (36.3%)		
	Gender	Female		Male			0.206
		238 (61.0%)		397 (65.0%)			
	Age	<5 years		≥5 years		+	<0.0001
		280 (70.4%)		351 (58.8%)			
	Habitat	Home		Displaced		+	0.038
		552 (65.1%)		112 (57.1%)			
**Injured**	Governorate	Aleppo	Idleb	Hamah	Lattakia	+	<0.0001
		27 (7.0%)	3 (0.8%)	1 (0.7%)	6 (7.3%)		
	Gender	Female		Male			0.406
		12 (3.1%)		25 (4.1%)			
	Age	<5 years		≥5 years		+	<0.001
		5 (1.3%)		32 (5.4%)			
	Habitat	Home		Displaced			0.82
		29 (3.6%)		8 (4.0%)			

**Table 4:** Crude Odds ratios for infectious disease and injury adjusted using multiple logistic regression analysis.

			O.R.	95% C.I. of O.R.		p-value
				**Lower**	**Upper**	
**Infectious disease** ^**a**^	**Origin** (reference: Aleppo)	Idleb	0.595	0.433	0.817	0.001
		Hamah	0.202	0.134	0.305	<0.001
		Lattakia	0.182	0.108	0.306	<0.000
	**Age** (reference ≥5 years old)	<5 years old	1.670	1.257	2.218	<0.0001
	**Displaced** (reference: home)	Displaced	1.010	0.709	1.438	0.958
**Injury** ^**b**^	**Origin** (reference: Aleppo)	Idleb	0.178	0.070	0.467	<0.001
		Hamah	0.168	0.039	0.721	0.016
		Lattakia	0.976	0.375	2.542	0.976
	**Age** (reference ≥5 years old)	<5 years old	0.181	0.070	0.467	<0.001
	**Displaced** (reference: home)	Displaced	0.961	0.417	2.215	0.925

C.I. confidence interval; O.R. odds ratio

(a) Likelihood ratio test (chi2): p<0.001, Hosmer–Lemeshow-test: p = 0.654, Nagelkerke’s R2: 0.135

(b) Likelihood ratio test (chi2): p<0.001, Hosmer–Lemeshow-test: p = 0.874, Nagelkerke’s R2: 0.133.

Injury was diagnosed in 37 patients (3.7%), versus 965 non-trauma primary diagnoses (96.5%). Trauma was significantly more present in Aleppo (n = 27/413;7.0%) and Lattakia (n = 6/82;7.3%), and among older children (n = 32/596; 5.4%), than in Idleb (n = 3/360;0.8%) and Hamah (n = 1/147;0.7%), or among children younger than five years old (n = 5/398;1.3%).

Trauma originated most frequently from accidental injury (n = 30; 3%); intentional violence was encountered by seven children (0.7%), all older than five. Only in Lattakia, intentional violent injury was more frequent than accidental trauma (p = 0.004).

The identified factors associated with the children's health problems and injuries ('governorate' and 'age') were then used in a multiple logistic regression analysis, as indicated in Table 4, revealing that children in Aleppo, and children younger than five have a significantly higher risk for infectious diseases; and that children in Aleppo and Lattakia, and children older than five have a significantly higher risk for injury.

The most common pre-existing comorbidities in the children were chronic respiratory diseases (n = 141; 14%) of which half (n = 69) concerned asthma, malnutrition related illness (n = 53; 5%), acute flaccid paralysis (n = 52; 5%), epilepsy (n = 40; 4%), eye diseases (n = 39; 4%), skin problems (n = 28; 3%), and mental disorders (n = 27; 3%).

Children aged under five represented 39.7% of the population (n = 398), with 62% boys (n = 247). Eighty-five (21%) were displaced, 7% (n = 24/369) lacked access to safe drinking water sources, 12% (n = 42/352) lacked access to appropriate sanitation facilities, 61% (n = 236/389) could not access paediatric healthcare providers, and 37% (n = 143/389) had an inadequate immunisation state.

The distribution of diagnostic categories indicates that 34% had respiratory diseases (n = 137), 23% digestive disorders (n = 90), 16% neurological disorders (n = 63), and 5/398 (1.3%) suffered from injuries.

## Discussion

In May 2015, four years after the start of the civil war, children in Northern Syria mostly suffered from respiratory diseases (29%), neurological disorders (19%) and digestive diseases (17%), while more than 4% of the children had been victim of violence or injury, and 2% of children suffered from mental problems.

The factors known to have an impact on the health of populations in conflict areas also apply to the children in this study: displacement and inadequate shelter, unsanitary living conditions, reduced access to clean water and safe food, insecurity, violence, and collapse of health services. This deterioration in living conditions results in an increased susceptibility to infectious diseases, injuries, malnutrition, exacerbation of non-communicable diseases, skin affections, neurological, and mental disorders [2,34].

The pattern and distribution of morbidity and disease resulting from this study can be compared to ranges found in the literature about other adult and paediatric IDP and RC populations originating from Syria, as demonstrated in Table 5. All of these ranges–except for pregnancy and birth–are proportionally higher than the rates indicated in the Global Burden of Disease rates for Syrian children in 2009 [12]. However, as there are no other studies available reporting on the health state of Syrian children in the last five years, the comparison summarised in this table has to be considered in the light of very different circumstances: most studies do not differentiate children from adults, all studies displayed were performed on Syrian refugees in other countries, and most studies handle different reporting templates or focus on specific diagnoses only, making comparison challenging and creating gaps in the table.

**Table 5:** Comparison of proportional ranges found in other studies (%).

situation	Respiratory	Eye & adnexa	Digestive	Neurological	Genitorurinary	Peripartum	Skin	Mental	Violence	Injury	Musculoskeletal	Circulatory	Infectious cases	NCD	Number
Syrian children (this study)	29	4.8	16.7	19.2	3.9	0.8	4.7	1.7	0.7	3.0	0.5	1.8	63.6	15.6	1002
ranges in the literature	1–56	2–10	1–22	0–9	0–13	1–4	0–10	0–38	0–22	1–39	0–15	0–22	3–90	2–50	
**GBD Syrian children** [12]	1.1		1.9	0.4	0.2	3.8	0	0.2	0	0.7	0	0.6	2.6	2.9	6850
**Refugee children in Jordan** [27]	36.3	2.0	7.7				6.4	0.4		6.1					1141
**Refugee minors in Germany** [29]	1		22		1		1						33		488
**Refugee minors in Germany** [33]	14.5		8.7	7.8	3.3			13.7				0	58.8	<2.0	102
Refugees in Turkey [31]	28	4	11		13		5	12			8	1		30	212
RC in Turkey [7]	11.6		1.4				0.1						13.1		982473
Refugees in Jordan [20]	11.0	10.0	14.9	6.0	3.0	4.0	9.6	1.3	3.4		9.0	22.0	<7.0	11–22	7642
Refugees in Jordan [42]								1.8							2526
Refugees in Jordan [43]	5.9										7.4	15.3		50.3	1550
RC in Jordan [44]								29.5						30.3	765
RC in Jordan [5]	29.7	2.2	5.6	0.1		2.4	3.5	1.3	0.5	4.3		5.3	72.1	21.8	694280
RC in Iraq [5]	56.4	3.3	11.7	0.2	4.7		4.8	0.5	0.2	2.2	0.1	3.2	89.6	7.4	127401
RC in Lebanon [5]	27.1		4.5	0.3	3.0	2.0	4.6	1.4	0.1	0.9		2.0	89.3	8.3	52060
MSF on the Balkan route [45]	25.0									30.0					3500
Refugees in Switzerland [19]	14.1						8.8	13.7		8.6	14.5	4.4	6.3		979
Refugees in Switzerland [32]			5.3	8.9				15.6	22.2	21.1	12.3		16.0	10.2	880
RC in Germany [46]	23.0	3.6	9.7	8.2	5.3	0.7	7.7	1.1			7.7	2.2	39.9	2.6	548
RC in Belgium [30]	35.5	1.7	7.8	3.6	2.5	0.7	8.6	1.9	0.9	12.5	6.1	1.8	48.6	7.0	3907
*Review mental health* [47]								*11–38*							*222 studies*
*Review ARI in crises* [48]	*3–55*														*36 studies*
*FH & IDP in Haiti* [38]	*16*.*5*	*4*.*2*	*10*.*7*	*2*.*5*	*6*.*8*		*4*.*0*	*2*.*5*	*0*.*0*	*38*.*7*		*1*.*7*	*37*.*8*	*3*.*3*	*2795*

GBD: global burden of disease, RC: refugee camp, NCD: non-communicable diseases, MSF: Médecins Sans Frontières, ARI: acute respiratory infections, FH: field hospital, IDP: internally displaced persons; italic = studies on IDP and refugees not from the Syrian crisis; bold = studies only considering children.

Infectious diseases were present in two-thirds of all children (n = 636), mostly of respiratory, neurological, and digestive origin. This reflects a well-known and earlier reported (Table 5) disaster-related threat resulting from the deterioration of living conditions in complex humanitarian emergencies [26,49]. Next to inadequate shelter and hygiene, these include overcrowding in IDP or refugee camps, facilitating the spread of respiratory infections, measles, food- and waterborne infections with diarrhea or polio-induced acute flaccid paralysis, and other communicable diseases [26,50]. Considering the documented inadequate immunisation state of Syrian children, this raises major concerns for otherwise preventable infections and epidemics developing among this vulnerable population in Syria and its neighbouring countries [17,34,50]. While geopolitical borders can delineate a conflict, they cannot contain the healthcare repercussions. This is demonstrated by the spill over of refugees prone to communicable diseases into Lebanon, Jordan, Iraq, and Turkey and may become a challenge for the herd immunity of all children in the Middle East and European regions now hosting numerous unvaccinated Syrian children [5,7,20,26–31,50].

When taking into account all primary and secondary diagnoses (Fig 1), the marked occurrence of respiratory diseases with upper and lower respiratory tract infections and asthma among the studied children becomes even more obvious (22%, 13% and 9% respectively). This corresponds with earlier reports from emergency medical teams in different types of natural and man-made disasters [5,27,30,31,38,45,46,48]. In addition to the aforementioned facilitating factors, the atomisation of rubble dust from destroyed constructions may also play a role, together with interruption of chronic treatment, especially in the occurrence of asthma cases [38]. The important proportion of lower respiratory infections is worrying, as these are among the top five known killers in vulnerable children [51]. Lack of pediatric healthcare and adequate treatment regimens undoubtedly contribute to this burden. Moreover, inappropriate and outdated treatment may introduce drug resistance in the population [49,52]. Due to limited access to laboratory and X-ray testing, detecting or confirming suspected cases of tuberculosis–entailing substantial morbidity and mortality in children–was not possible in this study. Although increased rates (up to 13/100000) were observed among Syrian refugees in neighbouring countries, Syria was reported to remain a low-incidence country [10,29].

Another concern is the presence of neurological infections in this study population, with 7.3% of the children featuring signs of meningitis, and 5.7% of acute flaccid paralysis (8% when considering also secondary diagnoses). Although the Middle East had been free of polio since the 1990s, the re-emergence of acute flaccid paralysis since 2013 has been described [25]. The polio immunisation coverage dropped from 91% in 2010 to 68% in 2012, subsequently over 7600 polio infections were reported, and the rise in incidence of acute flaccid paralysis to 4/100.000 children was confirmed in 2014–2015 [2,22,25,35]. Most cases were found in Deir Al-Zor, Aleppo and Idleb, and linked to the wild type poliovirus 1 from Pakistan (introduced by jihadi fighters), now contaminating unchlorinated water, food, and sewage in Syria [22,23]. The migration of people within and from a re-emerged endemic region carries the risk of spreading polio inside the country, but also to the neighbouring countries and to Europe [18].

In 2016, several cases of meningitis in Syria were reported to the WHO, most of which were of more benign viral origin. Given the situation, confirmation of bacterial meningitis was difficult for QRC. Aseptic meningitis/encephalitis may represent one of the key symptoms of poliovirus infection. Surveillance for enterovirus–as assurance of polio free status–in immigrants from Northern Syria, yielded 15% of faeces samples positive, 13% of which contained polio virus. The remaining samples were characterized as non-polio enterovirus [53].

Many diarrhoeal diseases were reported: mostly with watery (8%), but also with bloody stool (2%). Comparable vulnerable populations in conflicts demonstrated risks for typhoid fever and hepatitis A, both of which increased threefold in Syria in the first quarter of 2013 [12,22,49]. This is not only because of environmental deterioration, huddling in camps and weakened general health, but also because of failing surveillance, control and response of health systems [49].

Urinary tract infections (UTIs) have not been studied much in disaster settings or in war thorn areas, although emergence of urinary schistosomiasis has been mentioned [54]. The only available study about the Syrian situation, conducted at university hospitals in Aleppo in 2011, reports higher resistance levels of urinary detected microbiological agents than those reported by studies in neighbouring countries such as Kuwait and Iran. This was contributed to the misuse of third-generation cephalosporins and quinolones at Syrian hospitals and in the community [55].

Clinical malnutrition was the third most common diagnosis, considered in 13% of all children (4% as primary, and 9% as secondary diagnosis). In a report on Syrian refugees in Jordan, global acute malnutrition was relatively low unlike in most humanitarian emergencies [9]. Although most literature focuses on severe malnutrition, mild-to-moderate malnutrition may also be an underlying cause of death in children in complex emergencies, as it is in non-emergency settings [34]. Micronutrient deficiencies (iron, vitamin A and C, niacin, tryptophan, thiamine, iodine) are common in vulnerable and displaced populations [9,34]. In our study, clinical anaemia was diagnosed in 9% of the children (4.8% as primary and 4.2% as secondary diagnosis).

Young IDPs are also vulnerable for skin infections, as detected in almost 5% of the children in this study. This is again attributed to poor hygiene, sharing of combs with lice, exposure to parasitic vector-borne diseases like cutaneous leishmaniasis, and huddling in camps with spreading of scabies or air-borne viruses as measles and varicella, but also to a bad nutritional state, and Muslim ritual feet washing without drying [12,22,28,35,56]. Other skin disorders like xerosis cutis and eczema may be associated with the use of harsh soap, rough clothing, henna dying, and staphylococcal skin infections, but also with psychosomatic factors [56].

There were regional differences in disease patterns: in Aleppo, respiratory diseases (34%), injury (6%), and eye problems (6%) were proportionally more present than in the other governorates. Hamah children proportionally suffer more from acute mental illness (4%). Violent trauma (5%) and fatalities (1%) were more reported in Lattakia, as was neonatal illness (5%). The proportion of children with signs of infectious diseases was higher in Aleppo (76%) and Idleb (65%), governorates where digestive diagnoses were more common (21 and 19% respectively) than in Hamah or Lattakia (7% and 5%). This may be related to the lack of access to clean drinking water and sanitation in these regions [17].

Statistical analysis revealed that the risk for children to suffer from communicable diseases was higher in Aleppo (being the region longest under siege and with the highest rate of displacement, and for children younger than five years old as is usually seen in other disaster settings and in displaced populations) [4,17]. The risk of being injured was higher for children in Aleppo and Lattakia (both in the middle of the international conflict), and children older than five, including also child soldiers and children forcibly involved in adult tasks that put them at risk of being injured [17].

In this study, the non-trauma diagnoses (96.3%) outnumbered largely the trauma diagnoses (3.7%). This might be due to underreporting since children with physical trauma could not easily access specialized pediatric trauma care centres, which in turn may have led to higher mortality among these victims [1]. The proportion of injured children (6.1% adding primary and secondary diagnoses) is comparable to other reports, and to what is usually found in paediatric refugee populations [5,11,12,27]. Including also secondary diagnoses, 3.9% of the children in this study had accidental injuries and 2.2% suffered violence (0.3%) or had been victim of chemical agents (1.9%).

The proportion of mental disorders (6.9% when adding primary and secondary diagnoses) is lower in our study than observed among other children who experienced war [57,42]. The mental health of children in complex emergencies and armed conflicts has been studied, and might reach rates of up to 20%, although some reports showed contrarily a low incidence of psychological trauma [34]. A study covering 311 Syrian refugee children in Turkey on the other hand, reports that 79% had experienced the death of a loved one, 60% had been exposed to traumatic events like people being beaten or shot, 45% suffered from PTSD, 22% showed aggression, and 65% suffered from psychosomatic symptoms to a degree that seriously reduced the children’s level of functioning [57]. The healthcare providers in this study focused mainly on medical diagnoses, and may have underscored the mental problems of the examined children [2].

In accordance to earlier reports, children with chronical comorbidities mainly suffered from asthma (7%), and chronic respiratory illnesses (7%) [5,12,20,24,30–32,46,44]. Five per cent respectively had chronic diseases related to malnutrition, acute flaccid paralysis, epilepsy, eye disorders, and clinical anaemia. Pre-existing psychiatric diseases were present in 3%. Except for epilepsy, most of these illnesses could have been prevented, simply by providing regular and healthy meals, safe drinking water, access to specific paediatric or mother and child medical facilities (lacking for 64% of the children at the time of the study), immunisations, timely treatment, long-term follow-up, and health education [17].

### Limitations and strengths

There are several limitations to this study. First, due to a lack of a sound control population of Syrian children not affected by the civil war (hard to find during a disaster in a devastated country), it is impossible to link any increase of burden with certainty to the war. The study offers a point prevalence in May 2015, preventing measuring a difference with before the conflict. The sampling was done in four governorates only, as other regions were unsafe due to terrorist activities, avoiding generalisation of the results to the whole country. Although this study was thoroughly prepared in advance, with prospectively designed data sheets and well- trained and qualified data collectors, circumstances in the field were complex. The forms were filled out by different healthcare providers, possibly introducing sampling bias. A possible answer bias could be introduced by families fearing repercussions by authorities or rebel groups, when declaring or expressing suffering or discomfort of their children.

The strengths of the study are the considerable number of inclusions, the prospectively designed data registry records, and the extended information on diagnoses of children entangled in a complex humanitarian emergency.

### Interpretation

Four years of civil war in Syria led to circumstances with many children being displaced and living in substandard conditions. As in most complex humanitarian emergencies, inadequate shelter, limited access to water, food, and sanitation, together with worsening immunisation state and deficient pediatric healthcare provision contribute to the factors that put Syrian children at risk for malnutrition, communicable diseases, injuries and mental health problems, leading to increased morbidity and mortality [34].

More than two thirds of the children in Northern Syria suffer from infections, mainly of respiratory, neurological, digestive and dermatological origin. Many of these illnesses are known to be deadly in such a vulnerable population. Additionally, a large number of Syrian children will possibly bear the consequences of lifelong disability, physically as well as mentally, imposing an extra burden on the country's future.

Mitigation is possible by providing crucial interventions: fulfilling the basic needs of this population, timely surveillance and efficient case management, together with mass immunisations and health education [50,58]. As physicians we also urge to immediately cease attacks on hospitals, schools, and other critical civilian infrastructure, and to respect international humanitarian laws. Urgent coordinated and global action is needed to deal with this complex humanitarian emergency, and to prevent worsening of health threats to this generation of children in Syria, as they are the nation's main asset for the future.

### Generalisability

The findings of this study apply to children of the Northern Syrian region stricken by a civil war. Therefore, special caution must be taken in generalising the results of this study. Further research is needed to better assess the problems and needs of the affected populations in complex humanitarian emergencies.

## Supporting information

S1 File Patient data sheet (available in English and Arabic). (PDF)

S1 Fig Relative proportions of primary diagnosis categories, broken down per governorate (%). (PDF)
