# Supplementary material for: Diagnoses, infections and injuries in Northern Syrian children during the civil war: A cross-sectional study
Source: PLoS One. 2017 Sep 8;12(9):e0182770. doi: 10.1371/journal.pone.0182770 (PMC5590741; doi:10.1371/journal.pone.0182770)
Supplement: S1 File — (PDF) [file pone.0182770.s001.pdf]

Supporting Information

S1: patient data sheet (available in English and Arabic)

Patient Data Sheet / date of exam:……….

*Name of the patient (it will be added as a symbol eg. A1)………

*Age (years): ………….

*Gender: ……….

Male	
Female	

*Family status (ID: given symbol):….

Number of family members	Males:	Females:
Number of children		
	Yes	No
Father living		
Mother living		
Guardian (in case of loss of father/mother or both)		
Relation of the guardian to the child		

*Habitat:

	Yes	No
Separate own House		
Institution		
IDP camp		
Refugee tent		
Safe water source		
Appropriate sanitation		

*Nutrition status:

	Yes	No
Available		
Safe		
If Breast feeder available source		
Disease related to malnutrition		

If (yes) Please mention:………

*Unified Medical Home (Specific Health care provider):

Yes	
No	

If (yes), please mention the health care provider? ………

*Vaccination Status:

	Yes	No
Available		
Completed (for older children)		
Up-to-date (according to age category)		

If vaccinations are not completed (up-to-date), which vaccinations that child needs?

1-

2-

*Education:

Yes	
No	

If (yes) please mention the provider and in which grade:…………………………/…………………………

If (no) please mention quitted in which grade:…………………

***The most important health problem (patient's point of view):**

1-

2-

*** The most important health problem (Health worker's point of view):**

1-

2-

Other remarks:

Filled in by:

Check below one single main diagnosis (acute), check all secondary diagnoses and all chronic problems of this patient:

category	One main acute diagnosis	All Secondary acute diagnoses	All Chronic problems	DIAGNOSIS	Includes (case description)
no diagnosis				no medical diagnosis	social problem, attention seeker
respiratory				upper respiratory tract infection	ear, nose, throat, sinus, larynx infections, flu (upper ARI)
				lower respiratory tract infection	dyspnoea, and raised respiratory rate, signs of lower ARI
				asthma exacerbation	wheezing and/or respiratory oppression
eye & adnexa				eye disorder	eye infection and irritation
digestive				watery diarrhea/abdominal	loose stools, vomiting, abdominal pain, intestinal parasitosis
				bloody diarrhoea	loose stools with visible blood (suspicion of dysentery)
				malnutrition	clinical, weight/height >70% or MUAC <110/160 (child/adult)
				cholera	severe dehydrating diarrhoea/confirmed case non-endemic area
				jaundice	acute onset of icterus (skin, conjunctivae, urine)
neurological				suspected meningitis	fever and clinical signs of meningeal irritation
				flaccid paralysis	child ≥1 limb(s) (incl. Guillain Barré) or any age polio suspicion
				CVA, headache, convulsions	headache, convulsions, stroke, coma
genitourinary				sexual transmittable disease	suspected STD, vaginal infections with fluor, genital infection
				urinary tract infection	dys-, alg-, pollakisuria, with/without fever, flank pain, or + dipstick
				gynaecological disorder	irregular menses, breast problems, vaginal bleeding, abortion
peripartum				neonatal illness	newborns with problems
				neonatal tetanus	neonate not sucking/crying normally, rigidity, convulsions
				healthy new-born baby	healthy baby <3 weeks old
skin				skin infection	redness, pain, abcedation with signs of local infection
other				surgical cases other than trauma	hernias, swollen testicles, cysts, haemorrhoids,...
				fever of unknown origin	>37,5°C axillary or > 38,0°C rectal, without specific diagnosis
				malaria	confirmed or suspected malaria, simple or serious
				measles	fever and clinical suspicion of measles (vaccinated or not)
				clinical anaemia	history, pallor, weakness
				diabetes	diabetes as main problem, or crisis/ketoacidosis
				other	all that is not classified elsewhere
				intoxication	suspected or confirmed substance abusus
				neoplasm	suspected or confirmed oncological disease
mental				mental disorder	PTSD, insomnia, stress, suspicious aspecific complaints
violence				trauma from aggression	trauma due to intentional individual injury including rape
				CBRN	injury from chemical/biological/radiological/nuclear assaults
injury				accidental trauma	accidental trauma from incident, accident (non-violent trauma)
				acute wounds	non-intentional acute skin wounds, burns
musculoskeletal				musculoskeletal disorder	non-traumatic pain (muscles, back, pelvic belt, joints), rheuma
circulatory				hypertension/cardiac disorder	symptomatic hypertension, palpitations, angina pectoris
fatality				resuscitation	any condition requiring resuscitation of vital functions
				death	deceased patients
follow-up				follow-up wound dressings	follow-up of wound dressings and injections / vaccinations
				follow-up fractures & casts	follow-up old fractures & casts cases
				follow-up other	follow-up of chronic illness, other cases
