# Supplementary material for: Diagnoses, infections and injuries in Northern Syrian children during the civil war: A cross-sectional study
Source: PLoS One. 2017 Sep 8;12(9):e0182770. doi: 10.1371/journal.pone.0182770 (PMC5590741; doi:10.1371/journal.pone.0182770)

Supporting Information

S2 Fig: Relative proportions of primary diagnosis categories, broken down per governorate (%).

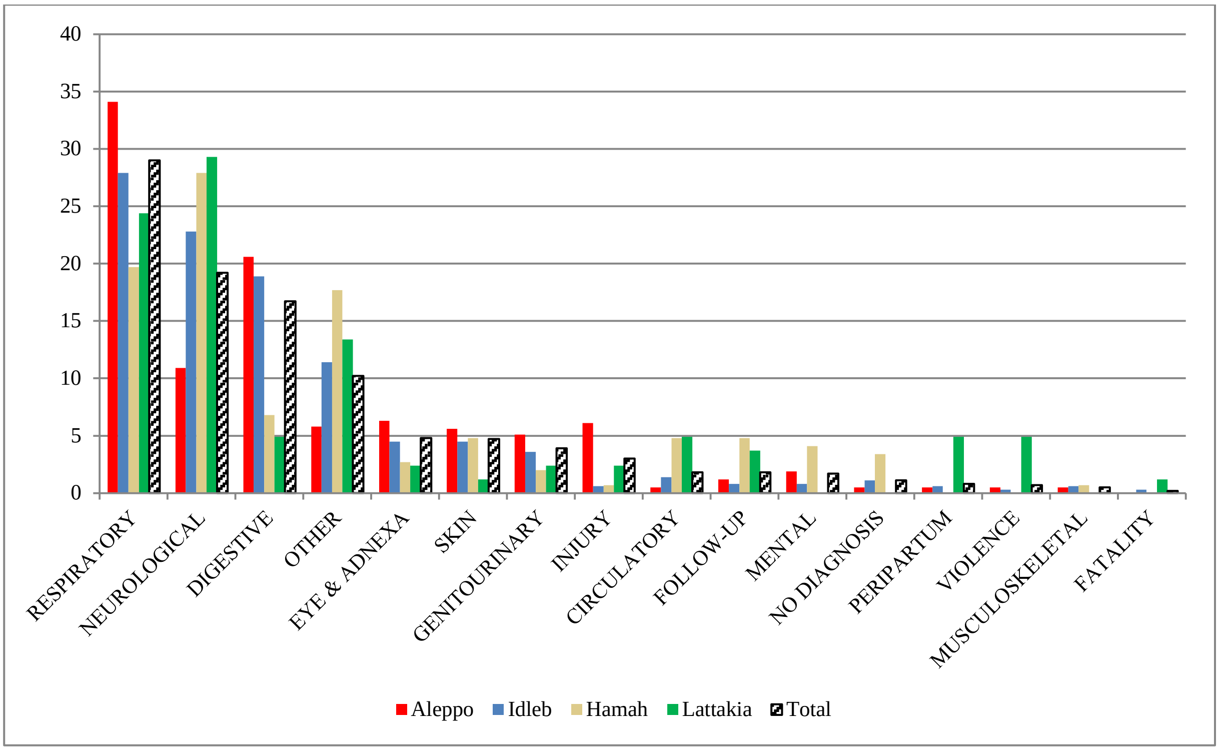

Supplement: S1 Fig — (PDF) [file pone.0182770.s002.pdf]
